# Introducing a bioelectrochemical method for highly selective enumeration of magnetotactic bacteria

**DOI:** 10.1038/s41598-020-65499-8

**Published:** 2020-05-22

**Authors:** Mina Memarpoor-Yazdi, Sara Haghighatian, Mohammad Mahdi Doroodmand, Abdollah Derakhshandeh, Maryam Sadat Moezzi

**Affiliations:** 1 Department of Chemistry, Shiraz University, Shiraz, Iran; 2 Department of Pathobiology, School of Veterinary Medicine, Shiraz University, Shiraz, Iran

**Keywords:** Biochemistry, Biological techniques, Biotechnology, Chemical biology, Microbiology

## Abstract

In this study, we employed an electrochemical (potentiometric) method to enumerate magnetotactic bacteria (MTB) during its coupling with iodometric titration to obtain a selective, precise and rapid counting system. Oxygen was considered as an important factor for the orientation and movement of MTB towards the magnet-modified indicator electrode. In the direct potentiometry, a linear correlation was detected between potentiometric response and dissolved oxygen (DO) concentrations. By the increase of the DO concentration, potential difference would increase in the range of 4.0 to 20.0 parts per million (ppm) at different pressure conditions. The reliability of the O_2_ bio-sensing feature provides a selective MTB-based cell enumeration methodology based on indirect potentiometric titration. Furthermore, a five-minute H_2_-purging resulted in an increase of potentiometric response sensitivity arising from the decrease in DO concentration of the electrolyte solution. Results were also investigated by zeta potential difference, which show the effect of charge density of MTB in presence of DO. Zeta potential was increased proportionally by addition of the MTB population. Regarding the reliability of the suggested method, data obtained by the designed system showed no statistical difference from those obtained by the most common procedure in microbiology for enumeration of bacteria, known as colony forming unit (CFU) method.

## Introduction

The detection and identification of bacterial species has been significant in a wide range of industries such as medicine, food technology/production, pharmacy, cosmetic, etc.^1^. Most of bacteria are nontoxic and beneficial for human health for instance, they can be employed for recovery of nutrients, nitrogen fixation, destruction processes, conversion of materials to energy and treat diseases. However, some of these bacteria are harmful and hence can be referred to as pathogenic bacteria^2^.

Having knowledge about population of bacteria in broth medium or a count of bacterial colonies in agar medium is significant for determining bacterial growth patterns and quantitative studies in different industries. Enumeration of the number of viable bacteria by formation of visible colonies on agar plates, known as colony formation, is a common method for determining bacterial viability^3^. On agar plates, the number of bacteria is often determined manually, which has some drawbacks such as low throughput, time-consuming, low accuracy, and it is tedious^4,5^. On the other hand, automatic numeration methods which are highly efficient, time-saving, with high resolution features and hence are often preferred as suitable alternatives to manual methods^6^. Although the resolution of major cell counting apparatuses like the hemocytometer^1^, coulter counter^1,6,7^, and flow cytometer^8^ can be affected by the cell population. Hence, by decreasing cell numbers to definite values, the accuracy of information is diminished^9^. Furthermore, these methods often need a purified population of target bacterium, often expensive equipment, and skilful operators. The alamar Blue is a designed assay to measure quantitatively the proliferation of different cell lines (human and animal), fungi, and bacteria, which has several benefits over usual cell counting assays^10,11^.

The colorimetric tetrazole 3-(4,5-dimethylthiazol-2-yl)-2,5-diphenyltetrazolium bromide (MTT) assay is based on the reduction of MTT to its respective formazan^12^. In this assay, purple dye appears which is then quantified using a photometer or a multiplate reader at the wavelength of 570 nm. Although this assay is often utilized to measure the proliferation and viability of mammalian cells, MTT and other relative materials can also be employed for measuring bacterial viability^7^. Another procedure to quantify bacterial growth is based on the measurement of the light scattering originating from the turbidity brought about by bacteria measured at 600 nm, referred to as optical density (OD_600_)^13^. This method is often used as a rapid and cost-effective technique to detect bacterial growth and throughout the culture in broth media. At a quick glance, however there are numerous methods for counting bacteria. Several investigations are being carried out to introduce highly selective, inexpensive, more accurate, and facile procedures. Among these, electrochemical sensing methods can be considered to overcome the limitations of culturing and biochemical procedures like sensitivity and specificity. Furthermore, they need lower technical knowledge than highly specific microbial or immunological methods.

MTB can easily be oriented towards the Earth’s magnetic field^14^. Magnetic and physical features of MTB make them valuable candidates in different fields including medicine like drug delivery^15^, treatment of infectious diseases, cancer therapy, and bioremediation^15–17^, cell separation^18,19^, enzyme immobilization^20^, biomineralization of metal ions^21^, waste water treatment, and for removing heavy metals^22–24^. However, although a wide variety of MTB’s applications have been reported so far, there are no reports available for using this type of bacteria in manufacturing a bacterial enumeration system.

The aim of our study is to present a highly selective, precise, and cost-effective method for enumeration of the MTB without requiring any time-consuming steps of bacterial purification. Our proposed method briefly employs an electrochemical (potentiometric) method, to detect the interaction of dissolved oxygen (DO) with the MTB. For measuring the DO parameter, iodometric titration is coupled with a potentiometric method. Based on the end point of titration (color of indicator and potential difference), the correlation of the magnetotactic bacteria population and potential differences are determined. Our results show that, the designed system can provide a simple and accurate chemical method for selective enumeration of magnetotactic bacteria without requiring their isolation from non-magnetotactic species.

## Results and Discussion

### Dual O_2_-H_2_ recognition biosensor based on the immobilized MTB

**Figure 1:**
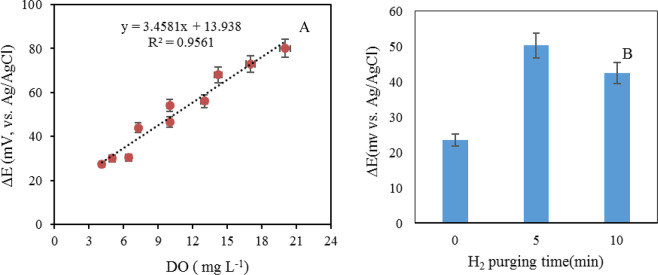
(**A**) Relationship between DO (concentrations ranged between 4.0–20.0 mg L^-1^) and the potentiometric response of MTB during useage of a two electrode system. Experimental condition: 10.0 mL Fe^2+^/Fe^3+^ (5.0 mg L^-1^), as well as the same volume of MTB-containing suspension at pH = 9.5 using Tris-buffer solution (0.02 mol L^-1^). Condition: O_2_/H_2_ purging time: 5.0 min. with flow rate of 400.0 mL min.^-1^. Electrode system: Ag/AgCl (Sat’d Cl^-^) as the reference electrode and rod modified (with magnet) as the indicator electrode at room temperature. The data are the average of three replicate analyses. (**B**) Effect of hydrogen purging time on the potentiometric response (n = 3).

Potentiometry has proved in many studies to be a powerful tool for designing biosensors^25,26^ and evaluate the capacity of microorganisms for biosensing purposes^27,28^. In our study, oxygen was considered as an important factor for movement and orientation of the MTB towards the magnet-modified indicator electrode. The relationship between DO and the potential difference in presence of MTB is shown in Fig. 1A. This shows that there is a linear correlation between the potentiometric gradient (ΔE) and the DO concentration.

Purging H_2_ gas for 5 min. increased the sensitivity of the electrochemical response. The effect of H_2_ gas was investigated by Thermogravimetric analysis (TG), which showed improvement of sensitivity. In TG analysis H_2_ absorbed /adsorbed by MTB indicates the main effect on performance of MTB. The maximum potentiometric response was obtained when DO concentration was set to 20.0 mg L^-1^, although salting-out effect has lowered this parameter in ecosystems^29^. Furthermore, studying the effect of different *pH* values demonestrated that the highest potentiometric sensitivity can be obtained at *pH* 9.0 prepared by Tris buffer (data not shown). Because of the presence of functional groups such as -OH and -NH_2_ in the structure of the Tris buffer as well as its buffer capacity (β), it behaved as the axillary complexing agent with cationic species such as Fe(II) and Fe(III). These cations are critical for the bacterial growth. They are mineralized inside the cell as Fe_3_O_4_. The optimum concentration of Fe(II) and Fe(III) to obtain a potentiometric response with maximum sensitivity was estimated to be 5.0 ppm.

Using direct potentiometry, the potentiometric response and DO concentrations showed a linear correlation, ranging from 4.0 to 20.0 ppm (Fig. 1A). Furthermore, in the optimum DO study, the effect of H_2_ gas on potentiometric response of the bacterium showed significant enhancement in different potentials during H_2_ purging. Figure 1B shows the increased sensitivity of the potentiometric response by H_2_ purging for 5.0 min. and the reversed effect by prolonging H_2_ purging time. This observation can be related to the decrease in the DO concentration of the electrolyte solution.

### Cell enumeration by the immobilized MTB

**Figure 2:**
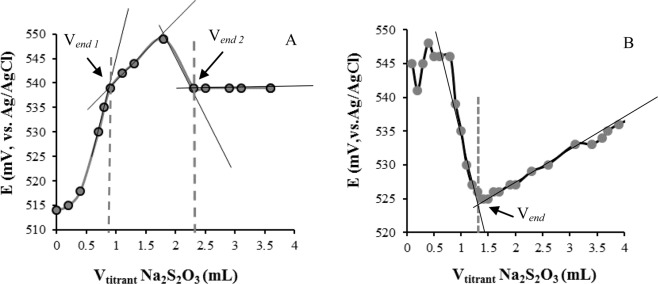
Titration of (**A**) MTB and **(B**) N_2_-treated MTB with Na_2_S_2_O_3_ (0.02 N), H_2_ purging time: 5.0 min. with flow rate of 400.0 mL min.^-1^. Starch was used as indicator. Electrode condition: Ag/AgCl as a reference electrode and rod modified (with magnet) as the indicator electrode. Titrant was added with the speed of 0.1 mL per 30 s.

The reliability of the O_2_ biosensing enabled us to introduce a selective MTB-based cell enumeration methodology using indirect potentiometric titration. In a typical experiment the titrant (Na_2_S_2_O_3_) is added to the analyte solution and the potential would begin to change until reaching titration endpoint (Fig. 2A). Two titration end points were detected belonging to free DO and MTB-interacted DO. However, only one end point was observed after deaeration and DO removal by N_2_ purging (Fig. 2B).

**Figure 3:**
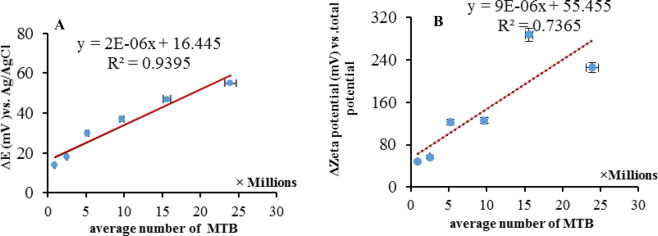
(**A**) Linear correlation between the average number of MTB and the potential difference during the potentiometric titration (n = 3). *ΔE* was defined as the potential gradient between *V*_end_ and *V*_initial_ of the titration. **(B)** Correlation between zeta potential gradient and different populations of whole MTB cells (n = 3).

Two independent endpoints were observed that correlated to *i*) the free DO in the electrolyte solution and *ii*) the DO adsorbed on the surface of MTB. Strong interaction of DO by MTB can probably lead to higher titrant consumption and subsequently observe two independent endpoints. Acceptable linear correlation was observed for the MTB population and the difference in potential (*ΔE*) and thus a calibration curve was obtained (Fig. 3A and Table 1).

**Table 1:** Correlation between the average population of MTB with *V*_*end*_ and *ΔE* in the potentiometric Winkler titration process.

Average number of MTB	*V*_*end*_ (mL)	*ΔΕ* (mV) vs. Ag/AgCl
84179 ± 18	0.80 ± 0.07	5.0 ± 0.2
92753 ± 35	0.50 ± 0.07	6.0 ± 0.2
965456 ± 26	0.37 ± 0.04	17.0 ± 0.2
1704485 ± 22	0.32 ± 0.05	10.0 ± 0.2
2229457 ± 25	1.00 ± 0.03	20.0 ± 0.1
2775321 ± 28	1.12 ± 0.05	18.0 ± 0.1
5696427 ± 36	0.65 ± 0.06	15.0 ± 0.2
5768324 ± 13	0.90 ± 0.06	30.0 ± 0.1
6584029 ± 35	0.43 ± 0.09	31.0 ± 0.1
6960743 ± 25	1.25 ± 0.06	31.0 ± 0.1
9679325 ± 23	0.61 ± 0.06	37.0 ± 0.1
15368282 ± 34	0.72 ± 0.05	47.0 ± 0.1
15783285 ± 24	0.62 ± 0.07	47.0 ± 0.1
23884063 ± 32	0.84 ± 0.08	54.0 ± 0.1

Three independent experiments were carried out and expressed. *ΔE* is the potential difference of titration.

This method can be presented as a simple, selective, rapid, and reliable chemical technique to number MTB indirectly during following the correlation between potential difference and the MTB population. In the presence of (non-MTB) no interference was detected in the enumeration of MTB population (Table 2).

**Table 2:** Selectivity of MTB-based counting system in the presence of non-MTB^a^.

Bacteria	Average population of the non-MTB	Titration response		Relative error percentage (%) based on *ΔE*
		*ΔΕ* (mV) vs. Ag/AgCl	*V*_*end*_ (mL)	
Control sample	67995218 ± 4	10.12 ± 0.28	1.22 ± 0.12	—
*Lactobacillus acidophilus*	368699293 ± 27	10.36 ± 0.26	0.48 ± 0.11	+2.37
*Lactobacillus gasseri*	197924249 ± 18	9.57 ± 0.25	1.21 ± 0.12	‒5.43
*Staphylococcus epidermidis*	258518225 ± 37	10.83 ± 0.21	1.36 ± 0.37	+7.02
*Escherichia coli*	7532834563 ± 218	10.82 ± 0.18	2.18 ± 0.52	+6.92

Three independent experiments were carried out and estimated *via* extrapolation of the calibration curve. Average population of the MTB used as the reference sample was 92753 ± 25 in all the tested samples. This method displayed a good selectivity towards MTB so that, after adding an excess population of different non-MTB (vs. the control sample), no statistical difference was detected. The presence of magnetosomes analog with MTB flagella will enable them to swim, orient and subsequently making mass transfer towards a special magnetic pole when they are subjected to an external magnetic field. This intrinsic feature will differentiate MTB mainly from non-magnetotactic bacteria and even from the cell debris of dead magnetotactic bacteria. This property makes them potential therapeutic carriers^30^. In addition, the affinity of the isolated MTB for adsorbing or absorbing O_2_ and H_2_ can be considered as one of the other distinguishable characteristics of these bacteria from the non-magnetotactic ones. In our study, these features were considered as the key factors for reliable MTB enumeration by the method used. Accordingly, the designed enumeration system will be able to differentiate magnetotactic bacteria from other non-magnetotactic ones. The reliability of this method was evaluated *via* comparison data obtained by calibration curve and number of MTB in the real sample. Maximum and minimum percentages of relative errors range from 0.15 to 10.53% (Table 3), which is acceptable for potentiometric methods.

**Table 3:** Estimation of MTB number in five synthetic MTB-based samples.

Sample No.	*ΔΕ* (mV) vs. Ag/AgCl	Real synthetic MTB population	Calculated population with the proposed method	Relative error percentage (%)
1	47.0	15783285 ± 28	15277500 ± 37	+3.21
2	28.0	5768324 ± 15	5777500 ± 41	‒0.15
3	44.0	12848517 ± 32	13777500 ± 26	‒7.23
4	37.0	9679325 ± 25	10277500 ± 25	‒6.18
5	31.0	6584029 ± 31	7277500 ± 16	‒10.53

It has been reported that the oxygen charge density is remarkably enhanced after coordination with other ion species^26^. Therefore, the charge density of the MTB was significantly changed after adsorption of O_2_. It is expected that, physicochmeical absorption/adsorption on the colloidal surfaces with high active surface area may provide extra charge density^31^. A fixed charge (Δq) was considered during the interaction between MTB and O_2_. In thermodynamics^32^, Δq is divided in two parts including static and dynamic contributions. The static part of Δq_static_ can be reported as dq_static_ = idt. The potentiometric technique is operated at open circuit conditions; therefore the share of this kind of electrical charge was negligible and was experimentally equal to zero^32^. Whereas about the dynamic part of Δq (i.e. Δq_dynamic_), this term is individually correlated to the capacitance behavior of MTB. This finding can be reported as dV = dq_dynamic_/dC, which is correlated to the surface charge (i.e. Zeta potential) of the microorganism^33^. In other words, reaction between titrant and O_2_ interacting with MTB changed the ratio of dq_dynamic_/dC, which is detected *via* their mass transfer (swimming) towards the indicator electrode. Eventually, these results were employed to measure the charge density (zeta potential) at open circuit conditions. To confirm this, the zeta potential of fresh MTB-containing solution was measured and compared to those estimated after reaching the endpoint of titration. As a result, a linear correlation was observed between MTB population and zeta potential difference (Fig. 3B). It was concluded that, the zeta potential difference was statistically affected by increasing the population of MTB and subsequently higher adsorption of O_2_.

## Conclusions

In the current study, a specified procedure for enumoration of magnetotactic bacteria was introduced by combining potentiometry and iodometric titration. Potentiometry is widely used as a powerful tool to assess the bio-sensing behavior of microorganisms. Using potentiometry, DO was detected as an affecting element on bacteria movement towards the magnet-modified indicator electrode and sunsequent potential difference so that a two or three-fold increase of DO concentration resulted in an enhanced accumulation of bacteria onto the elecrtode’s surface which was quantified by a two or three fold increase in potential difference. A significant increase of zeta potential difference was detected by enhancing MTB population. Furthermore, according to the TG analyses, a five minute H_2_-purching increased the potential difference to near two-fold displaying the effect of H_2_ absorption or adsorption behavior of bacteria on their movements and attachment onto the electrode’s surface. It has been concluded that, the introduced method provides a seletcive, fast, cost-effective, precise, and reliable tool for enumoration of magetotactic bacteria without the necessity of their isolation from other non-MTB.

## Methods

### Reagents and Materials

All reagents with purities more than 99% including starch, H_2_SO_4_ (96%) and also different salts including FeCl_3_.6H_2_O, FeCl_2_, Na_2_S_2_O_3_.5H_2_O, MnSO_4_.H_2_O, KI, NaOH, NaN_3_, trisaminomethane, different gasses (purity ≥ 99%), such as N_2_ and O_2_ were provided by gas cylinders and purchased from Linde (Germany). Reference electrode (Ag/AgCl/sat’d Cl^-^) and counter electrode (iron rode, diameter: 2.0 ± 0.1 mm) were all purchased from Metrohm (Switzerland).

### Design of the dissolved oxygen (DO) biosensor

The electromotive force (*E*_*emf*_) of the whole cell was measured using a potentiometer (Lutron Electronics Co Inc. 1986-01-22, Germany) and a two-electrode system including an iron rod (diameter: 2.0 ± 0.1 mm) as the indicator electrode and Ag/AgCl (Sat’d Cl^-^) as reference electrode. The indicator electrode was modified with a circular magnet (16.5 Tesla) with internal diameter, external diameter, and height of 1.5, 6.0, and 2.0 mm, respectively. High impendance noise was reduced significantly by empolying a thermal varnish around the indicator electrode. The electrolyte solution included 0.078 mM FeCl_2_ and 0.038 mM FeCl_3_.6H_2_O, Tris buffer (10.0 mM, optimum pH = 9.5), and the MTB solution (CFU: 3.2×10^7^, 10.0 mL). The volume ratio of Fe(II), Fe(III), and bacterial population was optimized to 1:1:2. Effects of H_2_ and O_2_ on potentiometric response of the bacteria were investigated by purging these gases independently into the electrolyte solution. Hydrogen was provided using a H_2_ generator (OPGU-1500 S, Korea) with a flow rate of 440.0 mL min.^-1^ for 5.0 min. Different concentrations of oxygen from 4.0 to 20.0 mg L^-1^ were purged into the electrolyte solution and then the resulting DO concentration was recorded using a DO meter (Lutron-5510, Lutron Electronics Co Inc. 1986-01-22, Germany). Concentration of H_2_ and O_2_ was optimized based on the one-at-a time method. To record the direct potentiometric response of the MTB on the indicator electrode, the *ΔE*_5min_ was monitored using the Visual Basic 6.0 (*VB*_6_) program.

### Construction of cell enumeration system

The obtained data from direct potentiometry was further confiremed by using the iodometry titration as an indirect method^24^. The *ΔE* of indirect potentiometry was calculated in the titration endpoint. In this method, the DO concentration is measured indirectly through an estimation of the end point of the potentiometric titrimetry. In this titration, 3.64 g MnSO_4_.H_2_O, 0.50 g NaOH, 0.10 g NaN_3_, 0.15 g KI and 1 mL H_2_SO_4_ (96%) were used as reagents, which were added to the analyte solutions (Fe(II) 0.078 mM and Fe(III) 0.038 mM and the MTB solution 1:1:2, V/V, in Tris buffer; 10.0 mM, optimum *pH* = 9.5), Na_2_S_2_O_3_ (0.02 M) and starch (average molecular weight: 342.30 g^.^mol^-1^) used as titrant and color indicator. The interfering effect of Fe(II) in titration was removed using sodium azide according to the established procedure^34^. Indirect potentiometry was carried out based the same procedure of direct method except for the addition of iodometric titration reagents, agitation and titration of the solution by Na_2_S_2_O_3_ until the end point. Furthermore, the selectivity of the presented method for exclusive enumeration of magnetic bacteria cells was investigated by using non-magnetic bacteria such as the gram-negative bacteria like *Escherichia coli* (*ATCC 10536*), *Salmonella enterica subsp. enterica* (*ATCC 13076*), *Pseudomonas aeruginosa* (*ATCC 9027*), the gram-positive bacteria *Staphylococcus epidermidis* (*ATCC 12228*), *Streptococcus vestibularis* (*ATCC 49124*), and *Lactobacillus amylovorus* (*ATCC 33620*) and the probiotic bacteria *Lactobacillus acidophilus* (KCTC 3164) and *Lactobacillus gasseri* (*ATCC 33323*) (all provided by the National Center for Genetic and Biological Reserves, Tehran, Iran). All these bacterial strains were tested as negative controls.

The net charge of the MTB surface is negative and can be balanced by opposite ions presented in the surrounding media^35^. Zeta potential has been considered as a useful technique to investigate the activity and mechanism of microorganisms^35,36^. In the current study, to determine the charge of the MTB cell surface in the presence of oxygen (100.0 parts per million, ppm), zeta potential was measured directly and indirectly. Direct method was carried out using a zeta potential analyzer (Svarovsky, Zeta Meter Inc., USA) via a two-microelectrode stainless steel plate (type: 316, 1.0×1.0 cm) with a distance of 2 cm. The capacitance current change was monitored using a galvanometer (United Scientific MGV002 DC Galvanometer) to reach the end point using a magnet (16.5 Tesla) and adding different populations of MTB and a digital multimeter (Fluke 177 TRMS digital multimeter, UK). The capacitance current was calculated by measuring the static and dynamic currents of the *RC* circuit (*R* = 100.00 ± 0.01 Ω and *C* = 5.00 ± 0.01 μF) using the multimeter (Fluke 177).
